# Supplementary material for: Are carbon emissions trading and green financial instruments synergistic? -Comprehensive quantitative research based on content analysis
Source: PLoS One. 2024 Mar 7;19(3):e0298601. doi: 10.1371/journal.pone.0298601 (PMC10919601; doi:10.1371/journal.pone.0298601)
Supplement: S1 Data — (ZIP) [file pone.0298601.s001.zip › Data/地级市Z_F工作报告.zip!/─┌├╔╣┼/│α╖σ╩╨/└┤╫╘╣1⁄2╓┌║┼í╛┬φ┐╦╩2╛▌═°í┐_2016.docx]

各位代表：

　　现在，我代表市人民政府向大会报告工作，请连同《赤峰市国民经济和社会发展第十三个五年规划纲要（草案）》一并审议，并请市政协委员和列席会议的同志提出意见。

　　一、2015年主要工作

　　2015年是十二五收官之年。在市委的坚强领导下，市政府带领全市各族人民，主动适应和引领经济发展新常态，认真落实四个全面战略布局，积极应对经济持续下行严峻挑战，强力攻坚重点工作重大工程，经济社会发展实现稳中有进，圆满完成了市六届人大三次会议确定的主要目标任务。全市完成地区生产总值1861.3亿元，增长8.1%;一般公共预算收入104.6亿元，增长6.7%;固定资产投资1272.1亿元，增长15.5%;社会消费品零售总额637.1亿元，增长8.3%;城乡居民人均可支配收入25195元和8812元，均增长8.6%。一年来，我们主要做了以下工作：

　　（一）综合施策稳定经济增长。发挥投资关键作用，实施5000万元以上重点项目638个，完成投资832亿元，220个项目竣工，赤峰至京沈高铁喀左站项目启动建设，国电投2×30万千瓦热电联产、中粮150万口生猪全产业链、赤峰商贸物流城、经锡高速公路、集通铁路增二线等重大项目加快推进。着力稳定企业生产，认真落实支持实体经济发展的系列政策措施，全年降低企业电费成本1.9亿元，减免企业和个体工商户税费17.1亿元，一企一策推动89户停产企业实现复产，新增规模以上工业企业30户，规模以上工业增加值增长9.1%。促进房地产业健康发展，实施契税补贴、降低首付比例、提高公积金贷款上限等措施，全市商品房销量增长15.9%。推动农牧业稳产增效，新增高效节水种植面积220万亩，粮食产量达103.2亿斤，6月末牲畜存栏2285万头只，规模以上农畜产品加工企业销售收入383亿元。引导金融机构加大信贷投放，推动企业开展多层次资本市场融资，全市直接融资企业达29家，金融机构贷款余额1163亿元,增长11%。

　　（二）多轮驱动促进产业转型升级。坚决淘汰落后产能，关停124家小矿山和小冶炼企业。鼓励工业企业技术创新，实施技改扩能项目35个、资源综合利用项目9个，完成脱硫脱硝除尘改造项目193个。壮大新型化工产业，推进13亿立方米煤制气、120万吨缓控释肥、4万吨季戊四醇等项目加快形成产能。围绕清洁能源输出基地建设，大力推进新能源项目，123万千瓦电力装机实现并网发电。推进赤峰制药、丹龙、蒙欣、艾克等医药企业退城入园，产能和装备水平大幅提升。大力培育电子信息产业，液晶显示屏产量达到1000万套，云计算孵化园正式运营，入驻企业48家。加快发展商贸物流、文化旅游等现代服务业，赤峰保税物流中心实现区域通关一体化，全市物流园区实现销售收入478亿元，旅游收入191亿元，第三产业增加值占GDP比重提高1.5个百分点。

　　（三）着眼长远增强可持续发展能力。抓住新常态下新机遇，认真谋划十三五发展，确定了总投资1.3万亿元的重大项目和重大工程。围绕做大做强赤峰经济开发区，启动赤峰城区东部经济带规划与建设，大力推进元电改接冀北电网、开发区自备电厂、铜产业园区、浩克新能源汽车重组等前期工作。围绕建设宜居城市，扎实推进智慧城市、绿色交通、综合管廊、河道治理、垃圾处理、污水处理等工作。围绕提升发展保障能力，谋划了千亿元的重大基础设施项目工程包，涵盖交通、水利、能源、电力、通讯等多个领域。围绕提升基本公共服务水平，加快推进市医院改造、职教城和健康养老园区建设等前期工作。围绕发挥政府投资的引导撬动作用，大力推广政府与社会资本合作模式，推出了千亿元PPP重点项目。

　　（四）注重实效推进改革攻坚。全年确定改革举措399项，形成改革成果214项。公布了市县两级政府权力清单，优化再造了工作流程，市本级减少审批环节706个、办理时限压减近60%。工商、质监、食药监实现三局合一，卫生计生机构改革全面完成。率先在全区推行三证合一、一照一码、全程电子化登记和电子营业执照，新增市场主体4.8万户，新增注册资本402亿元。进一步优化公共资源交易平台，实现交易额140.1亿元，同比增长27.7%。组建交通、工业、开发区基础设施三个投资公司，市级平台融资53.3亿元。颁布了全市河道管理办法和城镇集中饮用水水源地环境保护规划，开展了排污权有偿使用和交易试点。完成了左旗农村土地确权登记颁证和克旗草原确权承包试点，有序流转土地草牧场950万亩，宁城县、喀喇沁旗扩权强县试点稳步推进。完善了政府法律顾问制度，完成了救急难国家级综合试点工作。

　　（五）举全市之力推进十个全覆盖和扶贫开发两大工程。直面工程覆盖面广、任务艰巨的现实，把十个全覆盖工程作为最大的综合性民生工程，通过财政投入、金融支持、群众自筹等方式解决资金难题，通过加强领导、公开承诺、典型示范、严格考评等措施推动任务落实，全年完成投资115.5亿元，硬化街巷6244公里，改造危房5.4万户，新建饮水安全工程737处，全市1230个行政嘎查村实现了房路水电讯、文教卫保商十个全覆盖，1523个自然村同步建设。把十个全覆盖作为扶贫开发的主战场，将贫困村和贫困人口全部纳入覆盖范围，实施精准扶贫、精准脱贫，全年投入各类资金35亿元，13.2万贫困人口实现稳定脱贫。

　　（六）千方百计兑现民生承诺。认真落实《政府工作报告》中的民生工程和群众关切事项，全市民生支出321亿元，占财政支出比重达77.3%。新增城镇就业3.15万人，农牧民转移就业69.9万人。城乡基本养老保险参保人数160.6万人，基本医疗保险覆盖率95%以上，城乡低保实现应保尽保。为75.3万户低收入农牧户发放冬季取暖用煤补贴4.5亿元，为4259名低保家庭大学生发放就学补助1.5亿元。进一步完善中心城市和县城功能，新建改造供热、供气、给排水管网423公里，新增供热面积660万平方米，中心城区西大桥、新华大桥、西站大桥竣工通车，中环路全线连通。全年筹集公租房1024套，实施老旧小区改造110万平方米。总理关注的铁南棚改加快推进，当年完成投资13.4亿元。全市四项主要污染物排放减少4.2万吨，PM2.5下降12.8%，地表水达标率80%以上，中心城区空气质量优良以上天数比上年增加16天。教育、卫生、文化等各项社会事业取得新的发展和进步，林西县顺利通过国家义务教育均衡发展验收，全市19家县级公立医院全面推开综合改革，成功举办了中国国际微电影盛典，精神文明建设扎实推进，社会大局保持和谐稳定。

　　一年来，我们高度重视政府自身建设，以深化改革推进政府职能转变，切实改进工作作风，加快依法治市进程，加强党风廉政建设，扎实开展三严三实专题教育，深入开展不作为、慢作为、乱作为专项整治，自觉接受人大代表、政协委员和社会各界人士监督，政府治理能力和治理水平进一步提升。过去一年经济社会的健康发展，为十二五画上了圆满句号。

　　二、十二五回顾和十三五展望

　　十二五时期，面对复杂多变的宏观环境和经济持续下行的巨大压力，市政府紧紧依靠全市各族人民，与时俱进完善发展思路，凝心聚力破解发展难题，经济社会发展实现重大跨越，在全面建成小康社会的道路上迈出坚实步伐。

　　这五年，是综合实力显著增强的五年。全市地区生产总值年均增长10.8%,五年累计完成8230亿元，是十一五时期的2.1倍。一般公共预算收入突破百亿元,年均增长13.2%。固定资产投资累计完成4768亿元，是十一五时期的2.4倍。社会消费品零售总额年均增长13.3%，总量始终保持自治区前列。成功培育了1个千亿级产业、5个百亿级园区和3个百亿级企业，工业用电量净增22.9亿度，规模以上工业增加值年均增长14.7%。粮食产量连续4年超百亿斤，跻身全国产粮大市行列。牲畜存栏连续10年居自治区首位。年接待游客突破1200万人次，是十一五末的2.2倍。金融机构存贷款余额双双突破千亿元大关，较十一五末分别增加615亿元和697亿元。进出口总额累计完成38.4亿美元，是十一五时期的2倍。农博会升格为国家级展会并纳入农高会序列，成功举办了中国苜蓿发展大会等系列重大活动，成为国际牛羊肉产业大会、中国飞来者大会和中国国际微电影盛典永久举办地，国家级和自治区级品牌达103个，地区影响力和知名度显著提升。

　　这五年，是发展方式加快转变的五年。产业结构调整迈出坚实步伐，三次产业比由十一五末的16.3∶51.3∶32.4调整为14.9∶47.4∶37.7。新增新能源电力装机209万千瓦，装备制造、电子信息等战略性新兴产业产值近百亿元。万达广场等城市综合体引领传统商贸业提档升级，全市新增商业面积1062万平方米，电商物流、保税物流、陆港物流、冷链物流、金融物流等新业态蓬勃兴起，商贸物流业占三产比重提高2.1个百分点。1571工程基本完成，高效节水种植面积突破700万亩，农牧业综合机械化率和农畜产品加工转化率分别提高12个和3个百分点。投资结构不断优化，服务业投资、民间投资占比分别达到36.7%和67%。技改投资占工业投资比重达到25%以上。实行最严格的资源管理和环境保护制度，全面完成四项主要污染物减排任务，万元GDP能耗、水耗分别下降18.1%和47.5%，资源综合利用率提高4个百分点。五年引进高层次人才1600余人，新增自治区级以上科技创新企业和平台载体75个，取得各类科技成果和专利1978项。

　　这五年，是城乡面貌深刻变化的五年。新一轮城市总规修编完成，中心城市人口突破百万，建成区面积达105平方公里，比十一五末增加24平方公里。赤峰新区、桥北新区、松北新城建设突飞猛进，投入市政公用基础设施资金315.5亿元，城市综合承载和配套服务能力显著增强。全市9个县城建成区面积达到126.5平方公里，综合服务功能日趋完善。全市常住人口城镇化率由十一五末的41.2%提高到47.1%，人均城市道路面积和公园绿地面积分别提高到22.3平方米和16.9平方米，污水集中处理率由82.2%提高到89.3%,生活垃圾无害化处理率由60.2%提高到93.8%。 五年新增商品住宅2002万平方米，建成保障性住房1.9万套，实施老旧小区改造1005万平方米，改造棚户区、城中村6.1万户，完成农村牧区危房改造14.2万户。大力实施农村牧区十个全覆盖工程，完成投资165亿元，81.4%的行政嘎查村实现全覆盖，134.5万农牧民受益，建成了一批特色鲜明、生态宜居、文明和谐的美丽乡村。

　　这五年，是保障能力明显提升的五年。生态、交通、水利、能源、电力、通讯等重大基础设施累计投资超过1000亿元。完成生态建设面积1106万亩，新增国家和自治区级自然保护区3个，森林覆盖率提高到35.5%，荣获国家森林城市和自治区园林城市称号。新建改建各级公路1.1万公里,赤朝高速、赤承高速、赤凌一级路建成通车，全面融入国家高速路网，全市行政嘎查村通油路率达到79%。开工建设5条铁路，铁路运营总里程达到1101公里。完成玉龙机场改扩建，年旅客吞吐量近百万人次。建成饮水安全工程2661处，解决164万人饮水安全问题。气化赤峰工程全面启动。新建改造变电站69座，建成66千伏以上输变电线路3201公里。

　　这五年，是改革开放不断深化的五年。简政放权取得重大突破，市本级取消下放行政审批事项301项，比例达74.3%，全部取消非行政审批许可，废止和宣布失效红头文件507件。商事登记制度改革走在全区前列，民营经济实现健康快速发展，新增市场主体8.2万户，非公有制经济占比达56.1%，比十一五末提高了13.3个百分点。市场监管、文化广电、卫生计生等政府机构改革全面完成，事业单位分类改革有序推进，政府职能进一步转变。建立了全市统一的公共资源交易平台，全面实施财政资金预评审制度，节约财政资金70亿元。财税、投融资、农村牧区、生态文明、医药卫生、文化教育、城乡养老保险等重点领域和关键环节的改革取得重大突破，为经济社会发展注入了强劲动力。对外开放持续推进，京蒙对口帮扶合作不断深化，主动融入国家一带一路、京津冀协同发展和环渤海发展战略，一批实力雄厚的大企业大集团落户我市。与蒙古国后杭盖省、黑山尼克希奇市缔结为友好城市。五年引进国内区外资金3923亿元。

　　这五年，是民生和社会事业持续发展的五年。民生累计投入1299亿元,是十一五时期的2.7倍，占财政支出的比重始终高于70%。新增城镇就业14.4万人,农村牧区劳动力转移就业354.7万人次。城乡居民收入分别比十一五末增加了10852元和4263元，增速始终高于经济增长率。城乡基本养老保险制度全面建立，新型社会救助体系基本形成，困难群众、特殊群体、优抚对象生活得到有效保障。全民医保基本实现，城乡公共卫生和医疗服务体系进一步健全。各级各类教育加快发展，新建改造中小学和幼儿园641所，高中阶段免费教育全面实施。文化事业繁荣发展，群众文化生活日益丰富，红山文化和辽文化两大遗址进入世界文化遗产预备名单，我市进入全国文明城市提名城市行列。赤峰体育中心建成运营，圆满承办了自治区第十三届运动会。扶贫开发强力推进，投入资金95.7亿元，52.6万贫困人口实现稳定脱贫。国防建设、双拥共建、民族宗教、外事侨务、妇女儿童、残疾人、气象、地震、人防、统计、档案史志、慈善、红十字会等工作都取得了新成绩。

　　这五年，是政府自身建设全面加强的五年。法治赤峰建设扎实推进，六五普法全面完成，依法行政水平不断提高。平安赤峰建设成效显著，应急管理体系日趋完善，信访工作机制不断创新，社会治理能力明显提升，安全生产和食品药品安全形势总体平稳。市县两级全部公开了预决算，办公用房清理全面完成，因公出国、公务接待、公车使用得到严格控制，三公经费占财政支出比重逐年下降。主动向人大及其常委会报告工作，向政协常委会通报情况，累计办理人大代表建议610件、政协委员提案994件。政务公开、行政监察、审计监督工作成效明显，廉政建设和反腐败工作不断加强。

　　各位代表，过去五年取得的辉煌成绩，得益于自治区党委、政府和市委的正确领导，得益于市人大、市政协和社会各界的监督支持，更凝聚着全市各族人民的智慧和汗水。在此，我代表市人民政府，向全市广大干部群众，向人大代表、政协委员和离退休老同志，向所有关心和支持赤峰发展的社会各界人士，致以崇高的敬意和衷心的感谢！

　　在肯定成绩的同时，我们也清醒地认识到，赤峰欠发达的基本市情还没有根本改变，特别是在经济发展进入新常态的大背景下，我市经济社会发展面临的问题和困难仍然十分突出，政府工作面临的压力和挑战仍然十分严峻。主要是：经济总量小。产业发展不充分，传统产业比重大、链条短，资源优势未完全转化为经济优势；现代服务业和新兴产业培育不足、占比不高，拉动经济增长的作用有限；缺少大项目、大企业、大园区支撑，后续增长乏力。创新驱动力弱。思想解放不够，体制机制不活，科技投入严重不足，资金、技术、人才等要素制约明显，新理念、新模式、新业态的引进培育不够，发展动力仍需加快转换。城乡发展不协调。新型城镇化进程缓慢，城镇吸纳集聚人口的能力不强，城乡基础设施不完善，基本公共服务差距大。生态环保压力大。沙化治理、水土保持、区域绿化、低效林改造等生态建设任务重，破坏生态环境的现象时有发生；资源集约节约综合利用水平不高，水资源、土地资源、环境容量供需矛盾突出。对外开放程度低。与周边地区的联系不够紧密，区域合作助推发展的能力不足，融入京津冀、环渤海经济区发展尚未取得更多务实成果，借力一带一路战略更好地走出去尚未破题。改善民生任务重。贫困人口多，居民增收难，教育、医疗、社保等公共服务仍处在较低水平，与全面建成小康社会的目标要求还有不小的差距。政府自身建设仍需加强。有的地区和部门不适应形势和任务变化的要求，思维观念落后，方法创新不够，部分干部法治意识、责任意识和担当意识不强，不作为、慢作为、乱作为等懒政怠政行为仍然存在。这些都需要我们下大决心认真加以解决。

　　各位代表，过去五年的砥砺奋进，使今天的赤峰已经站在了一个新的历史起点上。十三五时期是全市经济格局调整的深化期，发展动力转换的关键期，全面深化改革的攻坚期，全面建成小康社会的决胜期，更是赤峰继往开来、转型发展、大有可为的重要战略机遇期。随着经济发展进入新常态，中央提出了一系列治国理政的新理念、新思想、新战略，为我们加快推进现代化事业提供了科学的理论指导和行动指南；全国全区经济长期向好的基本面没有改变，陆续出台了支持经济社会发展的系列政策措施，为我们做好各项工作创造了良好的宏观环境；国家加快实施供给侧结构性改革，全面打响脱贫攻坚战，深入推进一带一路、京津冀协同发展、环渤海经济区合作发展、振兴东北等老工业基地和西部大开发等战略，以及国内消费结构升级和发达地区产业加快转移，为我市调整结构、补齐短板、扩大对内对外开放带来了宝贵机遇。只要我们坚定信心、抢抓机遇、迎难而上、奋力拼搏，就一定能够把我市经济社会持续健康发展的良好态势长期保持下去。

　　根据市委关于国民经济和社会发展第十三个五年规划的建议，十三五时期全市经济社会发展的总体要求是：按照中央、自治区和市委工作部署，全面落实四个全面战略布局，全面践行创新、协调、绿色、开放、共享发展理念，积极适应和引领经济发展新常态，紧紧围绕建设国家有色金属冶炼加工基地、京津冀清洁能源输出基地、绿色农畜产品生产加工基地、特色文化旅游和生态休闲度假基地、国家级产业合作与转型升级示范区的发展定位，扎实推进结构性改革和发展动力转换，扎实推进做大总量和提质增效，扎实推进重点领域改革和民生改善，加快转变经济发展方式，加快新型工业化、信息化、城镇化、农牧业现代化和绿色化进程，加快开放合作和融入区域经济一体化步伐，努力形成经济新动力、发展新方式、产业新体系、城乡新面貌和开放新格局，确保到2020年实现全面建成小康社会的目标。

　　主要预期目标是：地区生产总值年均增长8%左右，一般公共预算收入年均增长7%左右，城乡常住居民人均可支配收入年均分别增长9%以上和10%左右，各项约束性指标达到自治区要求。

　　围绕落实上述总体要求和实现上述预期目标，今后五年市政府的工作将在以下六个方面持续发力、务求实效：

　　（一）坚持增量提质，壮大经济实力。注重发挥投资的关键作用，谋划实施一批重大项目，十三五期间规模以上固定资产投资增速不低于13%。加快新型工业化进程，推进资源型产业延长链条，发展非资源型产业，有色冶金、清洁能源、新型化工、农畜产品加工四大主导产业销售收入突破4000亿元，装备制造、现代医药、电子信息三大新兴产业销售收入突破500亿元，建材、纺织两大传统产业销售收入实现翻番。调整优化工业布局，依托赤峰经济开发区打造赤峰城区东部工业经济走廊，建成国家级千亿元园区，每个旗县培育一个百亿级园区。着力调整农牧业结构，大力发展高效、节水、特色农牧业，推动粮经饲统筹、农牧林结合、种养加一体、一二三产融合。实施3661工程，即到2020年农村牧区土地流转率、合作经济组织覆盖率、优质农畜产品就地加工转化率均达到60%，新增设施农业100万亩、优质紫花苜蓿100万亩、高效丰产经济林100万亩，粮食总产稳定在100亿斤以上，优质肉牛肉羊出栏1000万头只，优质生猪出栏1000万口。加快服务业提质增效，推动生产性服务业向专业化和价值链高端延伸、生活性服务业向精细化和高品质转变，把文化旅游业培育成支柱产业，建成国家级示范物流园区，第三产业增加值占GDP比重超过40%。大力发展非公有制经济，支持民营企业进入更多领域，引入非国有资本参与国企改革。支持县域经济发展，推进扩权强县，推动旗县区错位发展、分工协作，减少重复建设和无序竞争。

　　（二）坚持创新发展，推进动力转换。围绕提高发展质量和效益，改造提升传统动能，着力培育新动能。实施创新驱动战略，围绕产业链部署创新链，突出铜产业延伸加工和改造升级，建成国家铜冶炼加工基地。引进智能巴士、智能电网、大型机械、国家一类新药等生产线，做大做强新能源汽车产业。加快实施互联网+行动计划，促进互联网、云计算、大数据、物联网与经济社会发展各领域深度融合，与转型发展全面接轨。推进结构性改革，加快市场环境、产权制度、投融资体制、资源配置、人才科技等方面改革创新，注入发展新动力。创新拓展融资方式，通过设立发展基金、扩大信贷担保等措施，扶持优势企业和中小微企业发展壮大。推进国有资产管理体制改革，发挥现有投融资平台作用，组建新的资本投资公司，盘活政府存量资产，提升融资能力。大力推广政府与社会资本合作的PPP模式，激发民间投资活力。鼓励金融创新，扩大信贷规模，推进多层次资本市场直接融资，提升金融助推全市经济发展的能力。

　　（三）坚持协调发展，力促整体提升。尊重城市发展规律，改革城市建设管理体制，按照适用、经济、绿色、美观的方针，统筹推进智慧城市、海绵城市、园林城市、综合管廊、绿色交通、景观系统等建设，实现人文、低碳、智能发展。建成赤峰至京沈高铁喀左站铁路，力争开工建设城市轨道交通。加快市三区与喀喇沁旗协同发展，推动各旗县区新老城区和建制镇协调发展，基本完成棚户区和城中村改造，完善农牧业转移人口进城落户的政策措施，大幅提高常住和户籍人口城镇化率。推动市政设施和公共服务向苏木乡镇和重点村庄延伸拓展，促进农牧业人口就地城镇化。全面完成农村牧区十个全覆盖工程，健全基础设施和公共服务长效投入、管理机制，建设美丽宜居乡村。加快实施重大基础设施千亿元工程包，集中弥补综合交通网络不优、电力外送通道不畅、燃气气源保障不足、水资源相对短缺、宽带网络覆盖面窄等突出短板，增强城乡区域协调发展能力。推动物质文明和精神文明协调发展，力争建成全国文明城市。加强军地军民团结共建，巩固全国双拥模范城成果。

　　（四）坚持绿色发展，改善生态环境。围绕建设生态安全屏障，推进林业生态建设，到2020年完成营造林500万亩，新增文冠果木本油料林、防沙治沙综合示范林、樟子松基地和山杏林改造各100万亩，全市森林覆盖率达到36%以上。严格执行封育禁牧、休牧轮牧、休耕轮耕、退伐还绿、退耕还林还草等政策，推动生态环境通过自然恢复持续好转。加强环境综合整治，深入实施大气、水、土壤污染防治工程，推进工业污染源全面达标排放和城镇生活污水、垃圾处理设施、燃煤炉灶节能改造全覆盖，减量使用农业投入品，促进种养业废弃物无害化处理和资源化利用。科学规划、合理开发、有效整合矿产资源，促进探采选冶加一体化发展，提高综合开发利用水平。实行最严格的水资源管理制度，以水定产、以水定城，探索推行水权置换。强化约束性指标管理，落实能源、水资源消耗及建设用地总量和强度双控要求。深入推进节能减排，坚决淘汰落后产能。实行最严格的生态环境损害赔偿制度，落实环保机构体制改革，严格环保执法，推动环境质量持续好转。

　　（五）坚持开放发展，拓展合作空间。深度融入京津冀协同发展，推动各类产品进入京津冀市场，加快建设清洁能源输出基地，打造国家级生物天然气产业示范区。深化京蒙对口帮扶合作，积极承接发达地区整产业链和高新技术企业，建设国家级产业合作与转型升级示范区。主动对接国家一带一路战略，力促锡赤铁路开工建设，打通北接蒙俄、南下出海的国际大通道。积极参与区域共建，推动锡赤通经济区建设，努力在矿业经济、水煤组合、农畜产品加工、文化旅游等领域取得务实进展，打造区域发展新的经济增长极。抓住国家新一轮支持东北振兴的机遇，加强与东北经济区的对接合作。大力发展外向型经济，建设赤峰综合保税区，打造赤峰国际陆港。加强与蒙日韩、港澳台等国家和地区合作，不断巩固和扩大对外开放成果。

　　（六）坚持共享发展，增进民生福祉。实施脱贫攻坚工程，通过产业扶持、易地搬迁、转移就业、社保兜底、医疗救助、资产收益、培育新型农牧民等方式，到2020年贫困人口全部脱贫、贫困旗县区全部摘帽。促进就业创业工作，着力解决结构性就业矛盾，提高充分就业质量。千方百计增加城乡居民收入，确保与经济发展同步增长。健全多层次的社会保障体系，实施全民参保计划。全面提高教育质量，推动基础教育均衡发展，高度重视民族教育，大力发展学前教育，建成赤峰职教城。推动文化事业发展，提升文化软实力。推进群众体育、竞技体育和体育产业协调发展。大力发展健康产业，完善覆盖城乡居民的多层次医疗服务保障体系。加快发展养老服务业，满足群众多样化养老需求。全面实施两孩政策，促进人口均衡发展。推动社会治理创新，统筹安全生产、信访维稳、食品药品安全、社会治安等各项工作，营造和谐稳定的社会大局。

　　三、2016年重点工作

　　2016年是十三五开局之年。做好今年工作，必须深刻领会和全面贯彻中央、自治区和市委系列会议精神，善于寻找、发现、培育、把握新常态下的新机遇，落实五大政策，突出十个更加注重，着力推进供给侧结构性改革，全面落实三去一降一补五大任务。要下大力气化解过剩产能。正确处理巩固发展优势特色产业与化解过剩产能的关系，下决心淘汰落后产能、培育优势产能和压减低效产能。要扎实有序消化房地产库存。综合施策消化房地产库存，推进户籍制度改革，落实促进农牧业转移人口进城落户的具体办法和配套措施，释放刚性和改善性住房需求。要切实防范化解金融风险。做好政府存量债务置换、民间借贷监管、风险案件处置等工作，坚决守住不发生系统性、区域性风险的底线。要多措并举降低企业成本。落实好国家和自治区系列政策，在简政放权、正税清费、金融扶持、降低流通和电价成本等方面打出组合拳，为实体经济发展松绑减负、增添活力。要聚焦短板扩大有效供给。结合我市实际，提高基础设施投资的有效性和精准性，推进城乡基本公共服务均等化，促进资金资源向传统产业改造集中、向新兴产业发展集聚。

　　今年经济社会发展的主要预期目标是：地区生产总值增长7.5%，一般公共预算收入增长6.5%，规模以上固定资产投资增长12%，社会消费品零售总额增长10%，城乡常住居民人均可支配收入分别增长9%和10%，城镇登记失业率控制在4%以内，完成自治区下达的各项节能减排任务。具体实践中，要抓好以下四个方面工作。

　　（一）力推六大产业，构筑转型发展新格局

　　推动冶金产业延伸升级。围绕建设有色金属冶炼加工基地，支持市内企业与行业大企业大集团合作重组，实施一批技术先进、附加值高的精深加工项目，冶金产业年销售收入超过1200亿元。推进铜产业园区建设，实施赤峰云铜和金剑铜业搬迁技改扩能项目。加快中金岭南30万吨铅锌多金属冶炼等项目建设，确保远联钢铁150万吨宽带钢、东北特钢2万吨钼制品等项目达产达效。推动冶金与化工产业联动发展，就地转化利用硫酸等资源，力促大地云天磷肥一期竣工投产并启动二期建设，谋划实施一批新的氟化工、无机肥项目，实现产业链条式、园区循环式发展。推进风险探矿、深部探矿、外围找矿和矿产资源整合，组建矿业集团，开工建设有色产业综合体，为做强冶金产业提供有力保障。

　　推动清洁能源产业基地建设。抓好国电投热电联产、元易生物天然气等项目建设，实施大板工业园区智慧能源等项目，推动大唐煤制气一期Ⅱ系竣工投产，实现60万千瓦新能源项目并网发电。做好庆华煤基清洁能源、大唐煤制气二期、克旗120万千瓦抽水蓄能、大唐克旗2×66万千瓦火电、国电林东2×100万千瓦火电等一批重大项目前期工作。加快电力外输通道建设，力促元电改接冀北电网输电通道启动实施，推进赤峰至华北超高压外送通道前期工作。

　　推动现代农牧业加快发展。启动实施农牧业3661工程，抓好膜下滴灌成果巩固，完成设施农业、优质紫花苜蓿、丰产经济林各新增20万亩的建设任务。设立农牧业发展基金，支持新型经营主体发展，依托龙头企业带动、专业合作社引领、种养大户和家庭农牧场示范，推进土地草牧场依法有序流转，推广土地田保姆式托管服务，提高优质高效种养业比重。加快推进澳亚奶牛养殖、恒都肉牛屠宰加工、中粮生猪全产业链、凌志速冻薯条加工等项目，新建一批农副产品仓储物流、冷链物流、线上交易项目,着力发展粮食经济。做优牛羊肉、杂粮杂豆、果蔬等特色农畜产品，整合市域内资源，培育一批体现赤峰特色的绿色有机品牌。试行农牧业差异化考核，突出亩均畜均效益、综合机械化率、高效节水、冷链物流体系建设等指标，引导农牧业向规模化、标准化、产业化、集约化方向发展。

　　推动文化旅游业提质增效。成立文化旅游发展投资公司，打捆运营文化场馆、景区景点、基础设施，为产业发展注入资金动力。推进克什克腾、玉龙沙湖5A级景区创建，做好红山文化和辽文化两大遗址群申遗工作，开工建设辽上京国家考古遗址公园。建设文化旅游产品创意研发和展示展销基地，推出一批具有赤峰特色的旅游商品。推进智慧景区、自驾营地和旅游公共服务设施建设，加强旅游综合执法，提升旅游服务软实力。加大在主流媒体、黄金时段的文化旅游宣传推介力度，强化与周边地区深度合作，办好红山文化旅游节、微电影盛典等系列活动，提升赤峰旅游知名度和影响力。全年接待游客突破1400万人次，旅游收入达到220亿元。

　　推动现代物流业做大做强。编制完成全市物流业发展规划，明确目标定位、业态布局、发展重点和具体举措，增强物流业发展整体性和协同性。围绕打造国家级示范物流园区，启动联想集团冷链物流产业园建设，推进赤峰商贸物流城一期等项目和云商谷、内蒙跨境淘等电商平台建成运营，力促园区实现销售收入500亿元。加快新型基层供销合作体系和新网工程建设，推动农村牧区便民连锁超市及配送中心标准化建设，形成覆盖城乡、管理规范的物流配送网络。依托现代物流和电商平台，加快信息服务体系和配送体系建设，带动传统商贸业提档升级。

　　推动战略性新兴产业落地生根。加强与奇瑞、宗申等大企业大集团的战略合作，力促大型农牧业机械等一批装备制造项目开工建设，装备制造业销售收入达到60亿元。围绕建设赤峰经济开发区制药园区，积极引进和落地一批国家一类新药生产项目，加强与上海医药等行业领军企业的合作重组，推进赤峰制药尽快完成搬迁技改，确保普因医药抗生素等项目建成投产。加快发展生物化工产业，推动内蒙古伊品20万吨谷氨酸钠、科为博3万吨酶制剂等项目建成投产。积极发展互联网经济和电子信息产业，实施拓佳电子显示屏二期、云计算数据中心等项目，整合资源搭建全市综合性信息数据平台，创建国家电子商务示范城市。

　　（二）创优四个平台，打造持续发展新引擎

　　创优工业集中集聚发展平台。编制完成全市工业园区产业发展规划，明确各园区发展定位和产业布局，设立工业发展基金，出台发展飞地经济具体办法，推动项目、企业、产业向专业园区集中。组建园区发展融资平台，打包运营园区现有固定资产，吸引社会资本参与，破解基础设施投入不足等问题。围绕建设赤峰城区东部工业走廊，加快园区基础设施建设，开工建设赤峰经济开发区2×35万千瓦自备电厂，争取新增大用户直供电份额，以市场化运作方式配套建设综合服务基地，力促开发区销售收入800亿元。支持各地加大对工业园区投入，加快园区提档升级步伐，推动克旗工业园区进入百亿级园区行列。

　　创优投融资平台。着力推进投融资体制改革和金融创新，加强政府与金融机构合作，放大财政资金引导撬动效应，设立赤峰发展基金。大力推广PPP模式，细化工作流程，强化项目管理、前期评估和专家库建设，推动三座店水利枢纽向中心城区引供水工程、赤峰旅游综合服务中心、公共安全视频监控系统等项目落地实施，谋划推出一批新的PPP项目。理顺国有资产监管体制，加快组建文化旅游、水务、矿业、扶贫、建设等国有资本投资公司，推动国有企业去行政化。搭建推动企业直接融资的政府服务平台，支持企业到新三板和区域性股权交易中心挂牌，全市在资本市场直接融资企业力争新增6家、达到35家。完善市场担保体系，发展普惠金融。积极引进金融机构，扩大信贷投放规模，全市贷款余额突破1200亿元。

　　创优开放发展平台。围绕建设国家级产业合作与转型升级示范区，充分利用京津冀协同发展、京蒙对口帮扶合作等机遇，积极承接发达地区产业转移，全年引资650亿元以上。围绕融入国家一带一路战略，做好锡赤铁路前期工作，推进锡赤通经济区取得实质性进展。申建综合保税区，强化与锦州港、天津港合作，推进公铁海集装箱联运，搭建对外开放发展新平台。出台发挥保税物流中心功能、加快发展进出口贸易的政策措施，鼓励有实力的本土企业走出去，开拓进出口贸易、临空临港经济、国际仓储物流等外向型经济领域，全年进出口总额增长5%。

　　创优科技和人才支撑平台。加大科技投入，加强科技创新平台载体建设，在有色、药业、装备制造、农牧业等领域研发、引进和应用一批重大科技成果，为经济社会发展提供科技支撑。承办好2016中国北方农业科技成果博览会。启动赤峰职教城建设，推进城区一所高校、两所高职、五所中职有序搬迁入园，打造区域性职业教育综合改革试验基地和自治区重要技能型人才培训输出基地，将人文教育优势转化为人才智力优势。推进教育综合改革，促进义务教育均衡发展，加快教育信息化进程。加大财政投入力度，通过政府购买服务、PPP等方式引导社会资本参与，开工建设一批幼儿园。发挥我市基础教育资源优势，加快薄弱学校改造和学校标准化建设，出台支持社会办学的政策措施，为人才培养提供基础支撑。加快实施玉龙人才工程，创新人才引进、培养、使用和激励机制，为全市发展提供智力支持。

　　（三）打好五个攻坚战，开创城乡一体发展新气象

　　打好全面脱贫攻坚战。聚焦六个精准、五个一批，编制完成扶贫发展规划和易地扶贫搬迁规划，细化落实责任，层层分解任务，强化考核督查问责，大幅度提高减贫指标考核权重，确保脱贫工作取得实效。建立精准扶贫台账，实行贫困人口动态管理。加大资金整合投入力度，搭建市级投融资平台，拓宽扶贫资金筹措渠道，全年投入扶贫资金40亿元。实施好三到村三到户、金融扶贫、产业化扶贫等措施，开展雨露计划、光伏扶贫、旅游扶贫、电商扶贫、教育扶贫等精准扶贫工程，抓好定点帮扶、京蒙扶贫协作等社会扶贫工作，探索发展公益众筹扶贫，确保年内5.3万人稳定脱贫。

　　打好农村牧区十个全覆盖攻坚战。加大组织领导、资金投入、施工组织和工程管护力度，按照自治区的要求完成年内基本实现全覆盖的目标。着力打造十个全覆盖特色工程，实施农村牧区门户牌信息化管理，建设嘎查村便民服务体系，实现市县乡村办事大厅互联互通、一体化运行，打通联系服务群众最后一公里。在全市选取20个试点村集中发展农家乐、牧家游等休闲农牧业和乡村旅游，全年新增标准化经营户100家以上。健全农村牧区基础设施建设管理长效机制，研究出台具体办法，巩固工程建设成果。围绕美丽乡村建设，加快培育新型职业农牧民，实现变美与致富双赢。

　　打好新型城镇化攻坚战。推进中心城区130个重点市政工程项目建设，实施火花路大桥、银河大桥、铁南大街、英金桥续建等一批路桥工程，建设综合管廊10公里。启动中环路与环城高速连接路工程，规划赤峰城区至元宝山区和喀喇沁旗的轨道交通，开展公共自行车系统建设，打造中心城市50公里公交服务圈。启动海绵城市规划，开工建设智慧大厦。 推进中心城区绿亮美工程，实施生活垃圾焚烧发电项目和餐厨垃圾集中处理，推进西山生态产业园建设。加快社区综合体制改革步伐，综合施策提升社区服务能力。扎实做好物业管理、综合交通治理和市容市貌综合整治等工作。全市实施老旧小区改造190万平方米，启动解放西街等棚户区改造工程，改造棚户区和城中村7042户，确保总理关注的铁南棚改全面完成、7300户拆迁居民住上新居。推动中心城区市政设施和公共服务向周边村镇覆盖，实现一体化发展，今年选取7个重点镇开展试点。支持重点小城镇及周边村庄集中供暖、供气、给排水、垃圾和污水综合处理，促进农牧民实现就地城镇化。

　　打好生态环保攻坚战。深入实施大气和水污染综合整治工程，制定实施土壤污染防治工作方案。启动实施燃煤电厂超低排放和节能改造工程，取缔市区及旗县城关镇10蒸吨以下燃煤锅炉。加快林业生态建设和保护，年内完成营造林100万亩、重点区域绿化20万亩、水土保持综合治理105万亩。围绕创建国家园林城市，实施重点园林绿化项目53项，新增城市绿地6990亩。规划建设环中心城市50公里生态经济圈，启动中心城区河道综合治理工程，推进百公里锡伯河水利风景观光带建设。加快推进封育禁牧立法工作，依法加强自然保护区建设，重拳打击各类破坏生态环境行为。

　　打好重大基础设施建设攻坚战。全线开工赤峰至京沈高铁喀左站铁路、京通铁路电气化改造和叶赤铁路扩能改造，抓好集通铁路增二线等3条续建铁路。开工建设经棚至乌兰布统高速等5条公路，加快推进经锡高速等9条在建公路，新建通村油路2500公里。做好通用机场及低空飞行网络体系建设，力争林西支线机场开工建设，推进玉龙机场迁建前期工作。开工建设东台子水库和琥珀沟水库，推进境外调水工程前期工作。开工建设220千伏康家营输变电、文钟输变电工程，确保220千伏柏林输变电等19个电力工程竣工投运。

　　（四）夯实五大工程，巩固社会和谐稳定新局面

　　抓好就业创业工程。实施积极的就业创业扶持政策，增加有效就业供给，扶持劳动密集型产业、服务业和中小微企业发展，鼓励农牧民就地转移就业和外出务工人员返乡创业，重点解决好零就业家庭、高校毕业生、复转军人、城镇困难人员等特殊群体就业工作。全年新增城镇就业3.15万人，农牧民转移就业68.5万人，动态消除零就业家庭。推动大众创业、万众创新，设立创业发展基金，加强创业园和创业孵化基地建设，完成创业培训5100人、城镇就业技能培训1万人、农牧民技能培训2万人。

　　抓好社会保障筑底工程。扩大城乡居民养老保险覆盖面，启动全民参保登记计划。继续提高企业退休职工和城乡居民基础养老金、优抚对象抚恤金和城乡低保补助金标准。整合城乡居民基本医疗保险制度，合理确定城乡居民个人缴费比重，推进医保、医疗、医药三医联动，完善商业保险机构参与经办大病医保的具体实施办法，实现全民医保。继续办好三个一民生实事，完善各类救助体系建设，做好孤儿、残疾人、流浪乞讨人员等弱势群体保障工作，筑牢困难群体基本生活保障网。

　　抓好健康城市工程。推进公立医院综合改革，全面实施进一步改善医疗服务行动计划，努力解决人民群众看病难、看病贵问题。启动市医院改造和蒙东综合检验检测中心项目建设，加快肿瘤、妇儿等专科医院建设。加强卫生应急和急诊急救工作，实现120指挥调度系统全市联网运行。完善同级医疗机构检验、检查结果互认制度，推进医疗影像和临床检验中心建设，逐步实现大型医疗设备共用共享，提高资源利用效率。推广全科医生及乡村医生签约服务和远程会诊，实施分级诊疗，促进优质医疗资源向基层延伸。将区域卫生信息平台纳入智慧赤峰建设，完善居民电子健康档案、电子病历、人口信息等数据库，实现全市卫生计生服务一卡通。推进医养结合，建立居家养老、社区养老、机构养老等多层次养老服务体系。规划建设赤峰食品集中加工园区，实施食品药品安全专项治理，健全农畜产品追溯体系，保障人民群众舌尖上的安全。推动全民健身，倡导全民阅读，普及科学知识，完善相关配套服务设施，提升居民身心健康水平。

　　抓好文明创建工程。深入开展全国文明城市创建活动，扎实推进文明县城、文明村镇等多层次文明创建和最美家庭、道德模范、身边好人评选表彰，强化网络文明和创城公益宣传，全面提升市民文明素质和社会文明程度。规划建设市档案馆新馆，确保市青少年宫投入使用。推进社会信用体系建设，启动政务诚信、商务诚信、社会诚信、司法公信四大诚信工程。推动民族团结进步，依法管理宗教事务。深入实施文化下乡和文化惠民工程，加大非物质文化遗产保护力度，繁荣文艺创作，促进优秀民族文化传承保护和创新发展，建设文化强市。

　　抓好平安赤峰工程。推进法治赤峰建设，做好法律援助、人民调解、社区矫正、强制戒毒等工作，构建覆盖城乡居民的公共法律服务体系。加强社会治安综合治理，严密防范、严厉打击各类违法犯罪活动,切实增强群众的安全感和满意度。推进法治信访、责任信访和阳光信访，依法及时就地处理各类信访矛盾，妥善解决群众合理诉求。完善防灾减灾和应急救援体系，提高灾害防御和救助能力。严格落实党政同责、一岗双责、失职追责和企业主体责任、部门监管责任，强化安全监管力量，加大对矿山、化工、建筑、交通运输等重点行业和商场、学校、医院、车站、园区等重点领域的安全监管，坚决遏制重特大安全事故发生。

　　各位代表，让人民群众生活得更幸福，把赤峰建设得更美好，是我们的神圣职责和光荣使命。我们要坚持强化政治意识、大局意识、核心意识、看齐意识，在思想上行动上始终与党中央保持高度一致，严守政治纪律和政治规矩，全面、认真、坚决贯彻落实中央、自治区和市委的战略部署；坚持解放思想、更新观念，不墨守成规、不安于现状、不固步自封，始终用创新思维谋划发展；坚持直面问题、敢于担当，不推诿扯皮、不回避矛盾、不阵前退缩，努力用超常举措破解难题；坚持求真务实、苦干实干，不违背规律、不脱离实际、不铺张浪费，自觉用三严三实锤炼作风；坚持以人为本、执政为民，不做表面文章、不搞政绩工程、不搞形象工程,持续加强自身建设，着力打造人民满意政府。巩固党的群众路线教育实践活动和三严三实专题教育成果，深入开展学党章党规、学系列讲话，做合格党员学习教育，着力解决群众反映强烈的突出问题。强化宪法意识，树立法治思维，将政府活动全面纳入法治轨道，认真落实市人大及其常委会各项决议决定，自觉接受人大依法监督、政协民主监督和司法监督，加强政府内部层级监督、专门监督和审计监督，主动接受社会监督和舆论监督。深化行政执法体制改革，探索建立跨领域、跨行业综合执法。切实转变政府职能，大力推进简政放权，强化事中事后监管。推动政府机构改革，探索编随事转、财随事转机制，构建务实高效的政府组织体系。推进公共资源交易平台整合、政务服务标准化和电子政务建设，优化工作流程，推行服务承诺、首问负责、限时办结、网上审批、网上办事等制度，提高政府效能。加强政府绩效管理，严格追责问责，深化不作为、慢作为、乱作为专项整治，严厉惩治懒政怠政、失职渎职行为，打通政策落实最后一公里。严格落实党风廉政建设责任制，强化对重点领域和关键环节的监管，严肃查处违法违纪案件，严肃查处发生在群众身边的腐败问题。严格控制三公经费和一般性支出，全面推行公车制度改革，切实把有限资金更多地用在改善民生上。

　　各位代表，新常态孕育新机遇、新理念催发新优势、新起点开启新征程。让我们在市委的坚强领导下，励精图治，攻坚克难，锐意进取，为夺取全面建成小康社会伟大胜利而努力奋斗！
